# A Single-Center, Randomized Double-Blind Placebo-Controlled Study Evaluating the Effects of Poly-Gamma-Glutamate on Human NK Cell Activity after an 8-Week Oral Administration in Healthy Volunteers

**DOI:** 10.1155/2013/635960

**Published:** 2013-12-12

**Authors:** Kyung-Soo Kim, Tae-Young Lee, Jang-Hee Hong, Ahrom Kim, Sung-Jin Kim, Jai-Chul Choi, Moon-Hee Sung, Haryoung Poo

**Affiliations:** ^1^Department of Family Medicine, Seoul St. Mary's Hospital, The Catholic University, Seoul 137-701, Republic of Korea; ^2^Viral Infectious Disease Research Center, Korea Research Institute of Bioscience and Biotechnology, Daejeon 305-806, Republic of Korea; ^3^Clinical Trials Center, Department of Pharmacology, College of Medicine, Chungnam National University, Daejeon 301-721, Republic of Korea; ^4^Department of Microbiology and Molecular Genetics, Michigan State University, East Lansing, MI 48824, USA; ^5^Department of Advanced Fermentation Fusion, Kookmin University, Seoul 136-702, Republic of Korea; ^6^BioLeaders Corporation, Daejeon 305-500, Republic of Korea; ^7^Infection and Immunity Research Center, Korea Research Institute of Bioscience and Biotechnology, 125 Gwahak-ro, Yuseong-gu, Daejeon 305-806, Republic of Korea

## Abstract

A randomized double-blind placebo-controlled immunity study involving 99 healthy volunteers was performed to investigate the effect of poly-**γ**-glutamate (**γ**-PGA) on human natural killer (NK) cell activity in peripheral blood. The volunteers were randomly assigned to one of three groups and orally treated with solutions (25 mL) containing 0 mg (placebo), 250 mg (low dosage), or 500 mg (high dosage) of **γ**-PGA. Each volunteer took one dose every 12 hours for 8 weeks. Blood samples were drawn before the initial treatment and at the 4th and the 8th weeks of treatment. NK cell activity was assessed by measuring its degranulation, cytokine production, and cytotoxicity against the K562 cell line. Our results revealed that the cytotoxic activities of NK cells from the high-dosage **γ**-PGA group were significantly higher (*P* < 0.05 for all comparisons) compared to the low dosage and placebo groups at weeks 4 and 8 after the initial treatment. This increase in the NK cell activity among peripheral blood mononuclear cells (PBMCs) of healthy individuals was also confirmed *in vitro* (as assessed by the degranulation and cytokine production). These results suggest that the oral administration of **γ**-PGA induces a cell-mediated immunity by increasing the NK cell activity in humans.

## 1. Introduction

Poly-*γ*-glutamate (*γ*-PGA), which is naturally secreted from *Bacillus*, is a safe and edible polymer in which the **α**-amino and *γ*-carboxy groups of D- or L-glutamic acid are linked by isopeptide bonds [1]. We previously reported that oral administration of high-molecular-mass *γ*-PGA (average MW 2,000 kDa) isolated from *Bacillus subtilis sp. Chungkookjang* (a traditional Korean food) confers more significant antitumor effects than lower-molecular-mass (10 kDa) *γ*-PGA by inducing interleukin-12 (IL-12) and interferon-gamma (IFN-*γ*) production and activating natural killer (NK) cells in a mouse model system [2, 3]. These antitumor effects were elicited by the TLR4-dependent stimulation of immune cells (e.g., NK cells), activation of macrophages, and maturation of dendritic cells [3]. As part of their innate effector functions, NK cells possess a potent cytotoxic activity against tumor cells, virus-infected cells, and intracellular parasites [4–9]. NK cells destroy targets, including tumor cells, by various mechanisms, and they may be regulated by IL-12 secreted from activated macrophages or dendritic cells [10].

The effects of *γ*-PGA on the antitumor activity of human NK cells has not yet been reported. In the present study, we investigated the effect of oral administration of high-molecular-weight *γ*-PGA (MW 2,000 kDa) on the NK cell activity by examining peripheral blood mononuclear cells (PBMCs) from healthy volunteers who were treated in a single-center, randomized double-blind placebo-controlled study.

## 2. Materials and Methods

### 2.1. Reagents

*γ*-PGA molecules derived from *B. subtilis *(*Chungkookjang*), prepared as described previously [2], were kindly provided by BioLeaders Corporation (Daejeon, Korea) anddissolved in PBS. The number and weight-average molecular masses (M*n* and M*w*, respectively) and the polydispersity (M*w*/M*n*) of the *γ*-PGA molecules were measured by gel permeation chromatography using a GMPWXL column (Viscotek, Houston, TX, USA) and an LR125 Laser Refractometer (Viscotek). The polydispersity of 2,000 kDa *γ*-PGA was found to be 4.3. The compositional D/L ratio of *γ*-PGA was 60 : 40, as determined by High performance liquid chromatography (HPLC) after FDAA modification (for differentiation of D-glu and L-glu) of *γ*-PGA hydrolysates. Polyacrylamide standards (American Polymer Standard, Mentor, OH, USA) were used to construct a calibration curve. Anion-exchange chromatography was used to increase the *γ*-PGA content to >99% and decrease the polydispersity. To achieve complete solubility of the *γ*-PGA powder, we adjusted the pH of the solution to 6.8~7.0 with 5 N NaOH.

### 2.2. Study Designs and Drug Administration

Healthy volunteers (≥20 years) who met the selection criteria and were not on any therapy for physical or mental illnesses were enrolled in accordance with the guidelines of the Declaration of Helsinki. Approval was obtained from the institutional review boards of the Catholic University of Korea, College of Medicine (IRB approval number, KC10HSSI0064). Of the 102 participants, 99 met the trial criteria and were enrolled in the study. The study participants were randomized to three groups: 33 subjects (high-dosage) received 500 mg of *γ*-PGA powder dissolved in distilled water (DW, 22 mL) with maltitol (2.5 g); 33 subjects (low dosage) received 250 mg of *γ*-PGA powder dissolved in DW (22.25 mL) with maltitol (2.5 g); and 33 subjects (placebo) received maltitol (2.5 g) dissolved in DW (22.5 mL). All participants were orally treated twice daily for 8 weeks.

### 2.3. Preparation of PBMCs

PBMCs were isolated from heparinized venous blood samples using Ficoll-Hypaque density gradient centrifugation according to standard procedures [11]. For our *in vitro* assays, isolated PBMCs were cultured in RPMI 1640 (Invitrogen-Gibco, Carlsbad, CA, USA) supplemented with 10% (vol/vol) heat-inactivated FBS, penicillin (50 units/mL), and streptomycin (50 *μ*g/mL), at 37°C in a humidified atmosphere containing 5% CO_2_.

### 2.4. Tumor Cell Line

The human erythroleukemia cell line, K562, was cultured in RPMI 1640 (Invitrogen-Gibco) supplemented with 10% (vol/vol) heat-inactivated FBS, penicillin (50 units/mL) and streptomycin (50 *μ*g/mL) at 37°C in a humidified atmosphere containing 5% CO_2_.

### 2.5. Assaying the Cytotoxic Activity of NK Cells

The cytotoxic activity of NK cells against K562 cells, an NK cell-sensitive target cell line, was tested using a CytoTox 96 kit (Promega, Madison, WI, USA) according to the manufacturer's instructions. Briefly, K562 cells and PBMCs (including NK cells) were resuspended at a 30 : 1 ratio (PBMCs : K562) in RPMI-1640 containing 5% FBS, and incubated in 96-well round-bottomed plates for 5 h at 37°C. During this incubation, NK cell-mediated lysis of target cells caused lactate dehydrogenase (LDH) to be released into the medium. Spontaneous release of LDH from PBMCs or target cells was measured by a separate incubation of the respective populations. Maximum LDH release was measured by adding detergent to lyse all of the target cells (positive control). At the end of incubation, any cells that remained intact were centrifuged, and 50 *μ*L of supernatant from each well was transferred to a 96-well flat-bottomed plate and mixed with 50 *μ*L of fresh LDH substrate solution. The plate was incubated at room temperature for 30 min, and the reaction was stopped by the addition of 1 mol/L acetic acid. The resulting optical density (OD) was measured using a microplate reader at 490 nm. The percentage of cells exhibiting cytotoxic activity was calculated as: (OD of sample − [OD after spontaneous release of LDH from target cells + OD after spontaneous release of LDH from effector cells]) × 100/(OD after maximal release of LDH from target cell − OD after spontaneous release of LDH from target cells).

### 2.6. Analysis of NK Cell Activity following *γ*-PGA Treatment *In Vitro*

PBMCs (0.4 × 10^6^) from healthy donors were cultured in the presence or absence of *γ*-PGA (0.1 mg/mL) for 8 h and then stimulated with K562 tumor cells (0.2 × 10^6^) for an additional 7 h in the presence of monensin or brefeldin A, which detected surface CD107a and intracellular IFN-*γ*, respectively. Cells were stained with CD56 (Beckman Coulter, CA, USA), CD3, IFN-*γ*, CD107a (BD Biosciences, CA, USA), CD14 and CD19 (Biolegend, CA, USA) for flow cytometry, and CD56dimCD3-CD14-CD19-cells were gated as previously described [12].

### 2.7. Statistical Methods

The ITT (intent-to-treat) sample set was composed of volunteers who were tested for the main evaluation variable one or more times after the oral administration of *γ*-PGA or placebo, while the PP (per-protocol) sample set represented members of the ITT set who were fully tested and evaluated according to the clinical trial protocol. The data for the efficacy analysis were mainly based on the ITT set, whereas the PP set was used for the secondary analysis.

To evaluate the effect of *γ*-PGA on the cytotoxic activity of NK cells, we compared the cytotoxicity levels at baseline and treatment at weeks 4 and 8 and determined their averages, median standard deviations, minima, and maxima. In addition, we examined whether our results were normally distributed and used ANOVA and the Kruskal-Wallis test to calculate between-group differences among the average changes in cytotoxicity. When a significant difference was found, the data were compared further using Tukey's HSD multiple comparison test.

## 3. Results and Discussion

### 3.1. Study Population

**Figure 1:**
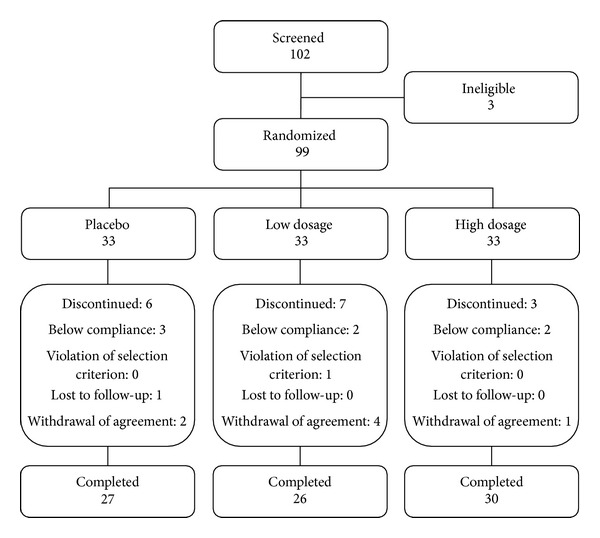
Distribution of study volunteers.

One-hundred-and-two healthy adults were recruited for this study between August 2010 and May 2011. Of the three subjects who did not pass the screening tests, the most frequent reasons for exclusion were that the individuals did not meet the selection criteria and/or were not assigned a random number due to an omission or administrative errors. The overall noncompletion rate was 15.7% (16 subjects); these subjects, who failed to meet the compliance requirements, violated the selection/exception criteria, became lost to follow-up, or withdrew agreement, were excluded from the PP analysis (Figure 1).

### 3.2. Demographics

The characteristics of the study participants in each treatment group are presented in Table 1. The numbers of male subjects in the groups were 5 (15.2%), 4 (12.1%), and 6 (18.2%) for the low dosage (250 mg of *γ*-PGA), high-dosage (500 mg of *γ*-PGA), and placebo groups, respectively. The numbers of female subjects in the groups were 28 (84.9%), 29 (87.9%), and 27 (81.8%) for the low dosage, high-dosage, and placebo groups, respectively. Overall, the number of female subjects was higher than that of male subjects, but the gender distributions did not differ significantly between groups. The average ages in years were 43.7 ± 13.5 (range, 20.0–57.0), 48.8 ± 11.5 (range, 20.0–64.0), and 45.3 ± 16.4 (range, 20.0–68.0) for the low dosage, high-dosage, and placebo groups, respectively. The differences in the average ages of the three groups were not statistically significant.

### 3.3. Efficacy Results

To evaluate the primary efficacy of *γ*-PGA treatment, NK cell cytotoxicities after 4 and 8 weeks of treatment were compared among the low dosage, high-dosage, and placebo groups. Based on the normality and Kruskal-Wallis tests, the between-group differences in NK cell cytotoxicity among the test subjects were determined to be statistically significant (*P* < 0.05), as described below.

**Figure 2:**
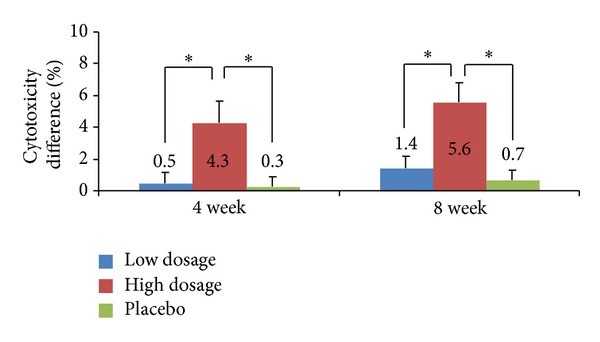
Lytic activity of PBMCs in the intention-to-treat (ITT) group. PBMCs were isolated from the test subjects who were treated with the low dosage of *γ*-PGA, high-dosage of *γ*-PGA, or no *γ*-PGA, and they were incubated with K562 cells for 5 h at an effector cell (PBMC) to target cell (K562) ratio of 30 : 1. Cytotoxicity was determined by the analysis of LDH release. *P* values are given compared to the baseline for the high-dosage, low dosage, and placebo groups. The results are given as the mean ± SD for donors; *P < 0.05.

*(1) ITT Group (Figure 2)*. A total of 99 ITT test subjects were divided into three test groups of 33 subjects each (low dosage, high-dosage, and placebo). Compared to the baseline, the cytotoxicities of NK cells from these groups were increased in average by 0.5 ± 3.5%, 4.3 ± 7.5%, and 0.3 ± 3.3%, respectively, at week 4. In a two-sided test with a significance level of 0.05, the difference in average cytotoxicity among the three test groups was statistically significant (*P* = 0.0174). Tukey's HSD multiple comparison tests also showed that the changes were significantly different between the low and high-dosage groups and between the high-dosage and placebo groups. At week 8, the average increases in NK cell cytotoxicity relative to the baseline were 1.4 ± 4.0% for the low dosage group, 5.6 ± 6.7% for the high-dosage group, and 0.7 ± 3.3% for the placebo group (*P* = 0.0013, two-sided test). Furthermore, the cytotoxicity levels differed significantly between the low and high-dosage groups and between the high-dosage and placebo groups according to Tukey's HSD multiple comparison test.

**Figure 3:**
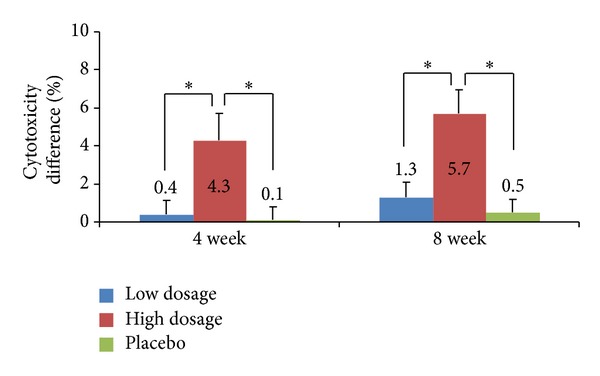
Lytic activity of PBMCs in the per-protocol (PP) group. PBMCs were isolated from the test subjects who were treated with the low dosage of *γ*-PGA, high-dosage of *γ*-PGA, or no *γ*-PGA, and they were incubated with K562 cells for 5 h at an effector cell (PBMC) to target cell (K562) ratio of 30 : 1. Cytotoxicity was determined by the analysis of LDH release. *P* values are given compared to the baseline for the high-dosage, low dosage, and placebo groups. Results are given as mean ± SD; *P < 0.05.

*(2) PP Group (Figure 3)*. After excluding the 16 test subjects whose reasons for exclusion were described in Section 3.1, a total of 83 test subjects were divided into the low dosage group (26 test subjects), the high-dosage group (30 test subjects), and the placebo group (27 test subjects). We then compared the average changes in NK cell cytotoxicity after 4 and 8 weeks of treatment. At week 4 of treatment, the cytotoxicities of NK cells showed average increases of 0.4 ± 3.6%, 4.3 ± 7.8%, and 0.1 ± 3.4% for the low dosage, high-dosage, and placebo groups, respectively, reflecting a significant difference among the groups (*P* = 0.0333, two-sided test). Tukey's HSD multiple comparison further revealed that there were significant differences in the cytotoxicity changes of the low versus high-dosage groups and between the high-dosage and placebo groups. At week 8 of treatment, the average cytotoxicity increases were 1.3 ± 4.1% for the low dosage group, 5.7 ± 6.8% for the high-dosage group, and 0.5 ± 3.5% for the placebo group, indicating a significant difference among the groups (*P* = 0.0014, two-sided test). Moreover, the cytotoxicity changes were significantly different between the high and low dosage group, and between the low dosage group and the placebo group according to Tukey's HSD multiple comparison test.

**Figure 4:**
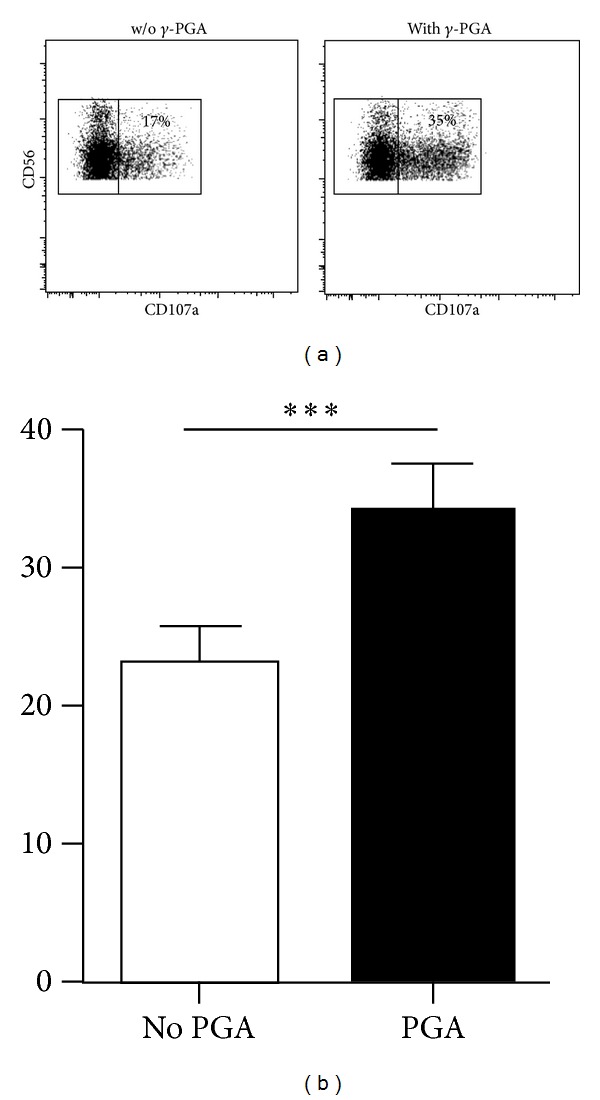
PBMCs from healthy donors were incubated with or without *γ*-PGA for 8 hours and then stimulated with K562 cells for an additional 7 hours. (a) Dot plots show CD56^+^CD3^−^ NK cells stained for CD107a from a representative donor. Inset numbers indicate the percentage of NK cells that displayed CD107a. (b) Bar graph shows the average percentage of CD107a^+^ NK cells from 13 donors; ***P < 0.0001.

**Figure 5:**
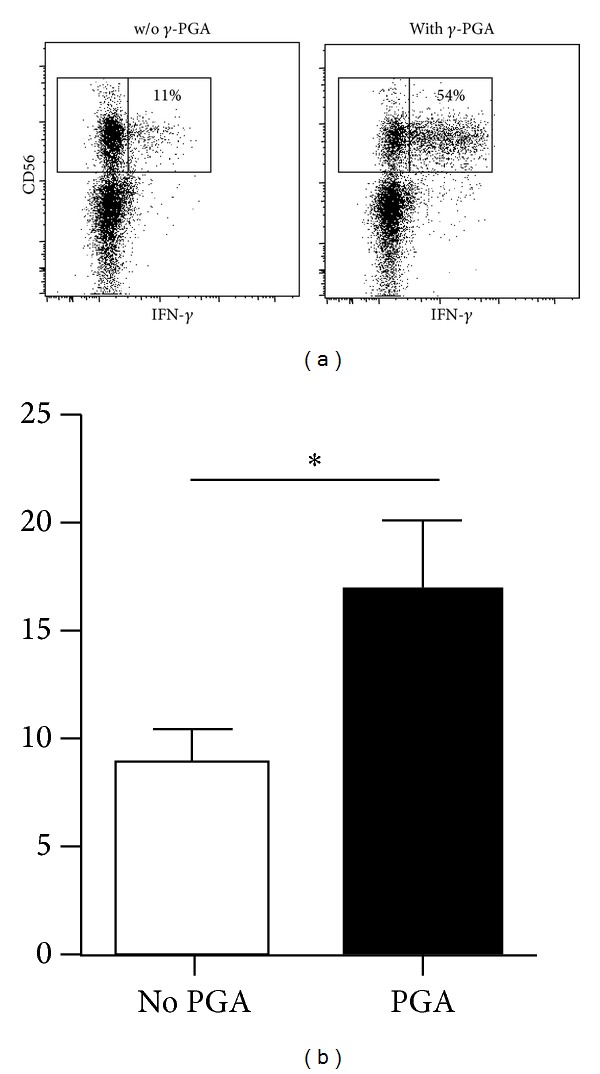
PBMCs from healthy donors were incubated with or without *γ*-PGA for 8 hours and then stimulated with K562 cells for an additional 7 hours. (a) Dot plots show CD56^+^CD3^−^ NK cells stained for intracellular IFN-*γ* from a representative donor. Inset numbers indicate the percentage of NK cells that produced IFN-*γ*. (b) Bar graph shows the average percentage of IFN-*γ* ^+^ NK cells from 16 donors; *P < 0.05.

We hypothesized that the increased cytotoxic effects of PBMCs from donors that received high doses of *γ*-PGA (in both the ITT and PP groups) might reflect increased NK cell proliferation in these individuals. To determine whether *γ*-PGA influences the effector functions of NK cells in addition to increasing the absolute number of NK cells, we cultured PBMCs from healthy donors *in vitro* in the presence or absence of 1 mg/mL *γ*-PGA for 8 h and then stimulated them with K562 tumor (target) cells. FACS analysis showed that a higher percentage of NK cells displayed CD107a (a surrogate marker for degranulation) in samples that were pretreated with *γ*-PGA compared to untreated samples (Figure 4). This analysis was done with at least 10 different donors, and the increase was consistent and statistically significant in almost all donors. Similarly, *γ*-PGA-treated NK cells produced higher levels of IFN-*γ* in the presence of K562 cells, compared to untreated samples (Figure 5). These results indicate that the effects observed in *γ*-PGA-treated donors are consistent with increased levels of NK cell cytotoxic activity and cytokine production.

NK cells, which are essential elements of the immune defense against pathogens and tumor cells, can modulate both innate and adaptive immune responses and are directly cytotoxic against virus-infected and tumor-derived cells. Many strategies have been investigated to enhance NK cell activity. Among them, *β*-glucan (isolated from *Ganoderma lucidum*) is an immunomodulator that is often consumed, especially among Orientals [13–15]. Furthermore, a significant proportion of cancer patients takes complementary medical therapies while receiving conventional anticancer treatments [16–18]. Thus, we herein sought to establish the immunopotentiating function of *γ*-PGA in humans. We report for the first time that both IFN-*γ* and cytolysis are upregulated in *γ*-PGA-treated NK cells *in vitro*. Also, a significant and consistent pattern of enhancement was observed in healthy volunteers treated with *γ*-PGA. Thus, it may be possible to use *γ*-PGA treatment to diminish tumors in some cancer patients.

### 3.4. Safety Assessment

The treated and untreated donors did not show any significant difference in abnormal diagnoses or clinically relevant changes in laboratory parameters, vital signs, or other safety parameters.

## 4. Conclusions

In conclusion, this randomized double-blind placebo-controlled trial revealed that a high-dosage of *γ*-PGA (naturally secreted from *Bacillus subtilis sp. Chungkookjang*, a traditional Korean food) significantly increased the cytotoxic activity of NK cells compared to the placebo control. This increase in NK cell activity was also confirmed *in vitro*, measured in terms of degranulation and cytokine production. These results collectively suggest that oral administration of *γ*-PGA induces cell-mediated immunity by increasing NK cell activity in humans.

## Figures and Tables

**Table 1:** Demographic characteristics of the study subjects.

Category	Low dosage	High dosage	Placebo	Total	*P* value
	*n* = 33	*n* = 33	*n* = 33	*n* = 99	
Sex					
Male	5 (15.2%)	4 (12.1%)	6 (18.2%)	15 (15.2%)	
Female	28 (84.9%)	29 (87.9%)	27 (81.8%)	84 (84.9%)	0.7900
Age					
Mean ± SD	43.7 ± 13.5	48.8 ± 11.5	45.3 ± 16.4	45.9 ± 14.0	
Median	51.0	53.0	54.0	53.0	
Min~max	20.0~57.0	20.0~64.0	20.0~68.0	20.0~68.0	0.2281
